# Organic acid toxicity, tolerance, and production in *Escherichia coli *biorefining applications

**DOI:** 10.1186/1475-2859-4-25

**Published:** 2005-08-25

**Authors:** Tanya Warnecke, Ryan T Gill

**Affiliations:** 1 Department of Chemical and Biological Engineering, UCB424/ECCH120, University of Colorado, Boulder, CO 80309, USA

## Abstract

Organic acids are valuable platform chemicals for future biorefining applications. Such applications involve the conversion of low-cost renewable resources to platform sugars, which are then converted to platform chemicals by fermentation and further derivatized to large-volume chemicals through conventional catalytic routes. Organic acids are toxic to many of the microorganisms, such as *Escherichia coli*, proposed to serve as biorefining platform hosts at concentrations well below what is required for economical production. The toxicity is two-fold including not only pH based growth inhibition but also anion-specific effects on metabolism that also affect growth. *E. coli *maintain viability at very low pH through several different tolerance mechanisms including but not limited to the use of decarboxylation reactions that consume protons, ion transporters that remove protons, increased expression of known stress genes, and changing membrane composition. The focus of this mini-review is on organic acid toxicity and associated tolerance mechanisms as well as several examples of successful organic acid production processes for *E. coli*.

## Review

### Biorefining Platforms

**Figure 1:**
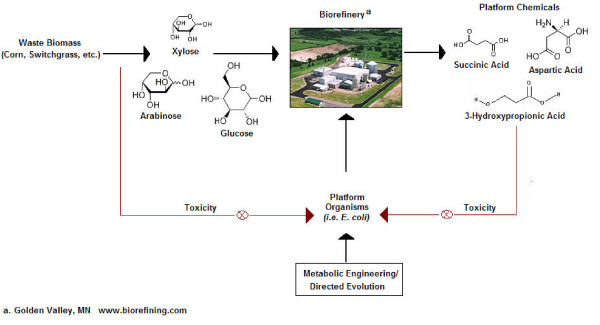
Conceptual model of toxicity in biorefining applications. Sugars are extracted from waste biomass for use as feedstock for platform organisms in a biorefinery. Metabolically engineered microorganisms convert sugars into valuable platform chemicals that are then further derivatized to large-volume chemicals. Product and feedstock toxicity are observed, thus limiting productivity of biorefining applications.

Biorefining promises the development of efficient processes for the conversion of renewable sources of carbon and energy into large volume commodity chemicals. It has been estimated that such bioprocesses already account for 5% of the 1.2 trillion dollar US chemical market [1], with some projecting future values of up to 50% of the total US chemical market generated through biological means. While the attractiveness of such bioprocesses has been recognized for some time [2,3], recent advances in biological engineering and associated sciences [4-15], several biorefining success stories [16-18], and instability in the price and future availability of oil [19], have collectively reinvigorated interest in the large scale production of chemicals through biological routes. Nevertheless, many challenges still remain for the economical bio-production of commodity chemicals. Such challenges encompass the need to not only inexpensively convert biomass into usable sources of carbon and energy but also to engineer microbes to produce relevant chemicals at high titers and productivities while minimizing the generation of byproducts that might foul downstream processes [1,20,21]. One model for addressing the latter of such challenges involves the generation of platform organisms that can be easily engineered and re-engineered to produce a variety of building block chemicals that are amenable to conversions to higher value products via traditional catalytic routes (see Figure 1). Although chemical pretreatment of raw materials impairs viability of platform organisms, this review will focus on product toxicity issues associated with the production of organic acids in *E. coli *(for further information on sugar extraction from raw materials see Zaldavar, *et al*. [22] and Knauf, *et al*. [23]).

The US Department of Energy (USDOE) recently released a prioritized list of building block chemicals for future biorefining endeavors. Priority was assigned based on the projected value of the platform chemical and potential derivatives as well as what technological developments were required for the production of the chemical and associated derivatives [21]. The report emphasized the importance of organic acids to the future of biorefining efforts (eight of the top twelve chemicals were organic acids, see Table 1 in additional file 1). The USDOE is not the first to recognize the importance of organic acids. In fact, there is a rich literature describing microbial production of organic acids [17,20,24,25], including several successful commercial bioprocesses [26-28]. Product toxicity is one of the primary challenges in the development of organic acid bioprocesses based on the use of platform host organisms, such as *E. coli*. In particular, while *E. coli *is known to survive very high concentrations of acids (pH = 2) when passing through the mammalian stomach, *E. coli *are surprisingly acid sensitive in exponential phase when cultured planktonically [29,30]. Moreover, undissociated organic acids, which pass freely through the outer and plasma membranes of *E. coli *[31,32], dissociate upon entry into the slightly alkaline cytoplasm releasing protons that lower internal pH (pH_i_) and anions that specifically inhibit different aspects of metabolism resulting in impaired growth [33-35]. Titers and productivities of 50–100 g/L and 2–3 g/L·hr are expected for the economical manufacturing of most building block acids by fermentation. The pKa values range from 3–5 for these organic acids, which would result in a pH reduction to around 2.0 for titers of 50 g/L. This highlights a key challenge in the metabolic engineering of organic acid production hosts. That is, high titers result in the addition of protons to the culture, which either result in a decreased pH or the addition of large volumes of base titrant. At low pH, organic acids are undissociated, thus they pass freely through the membrane and inhibit growth. At high pH, the process is less efficient due to base requirements and because export of the organic acid cannot proceed by free diffusion alone (for a more detailed discussion of organic acid export issues see Van Maris *et al*. [36]). What is desired, therefore, is a platform organism that not only produces high levels of organic acid chemicals but also is tolerant to any associated toxicity.

Many microbes are capable of producing platform chemicals by aerobic and anaerobic fermentation processes [22]. L-lactic acid has traditionally been produced by lactic acid bacteria. Although many lactic acid bacteria strains have been studied extensively [37], the ability to produce optically pure L-lactic acid is hampered by the presence of both L and D lactate dehydrogenase genes [38]. Pure L-lactic acid must therefore be produced via another pathway, as the racemic acid product is not useful for downstream conversion into polylactic acid. A number of other microorganisms have been used for industrial fermentation of several of the building block organic acids identified in Table 1. Large scale production of amino acids has been accomplished in *Corynebacteriumglutamicum *[39], succinic acid has been produced by *Actinobacillus succinogenes *[40], and itaconic acid production has been carried out with *Aspergillus terrus *[41]. While successful, the future application of these organisms as platform hosts is limited when compared with *E. coli*. *E. coli *is advantageous as a platform host because it is the most well characterized model organism, it has been used in recombinant processes for over 20 years, there are a wide variety of good genetic tools, and it is sensitive to many antibiotics used in genetic engineering efforts [42]. Moreover, the completion of the *E. coli *genome sequence has already enabled many functional genomics studies and proven useful in metabolic engineering efforts [43]. Finally, *E. coli *grows quickly in minimal media and maintains the ability to metabolize both 5 and 6 carbon sugars, which is a specific advantage over the use of industrially relevant yeast strains [22]. This mini-review will describe the basic mechanisms underlying organic acid toxicity and associated tolerance pathways in *E. coli *followed by a short discussion of several metabolic engineering strategies employed for the production of organic acids in *E. coli*.

### Organic Acid Toxicity in *E. coli*

**Figure 2:**
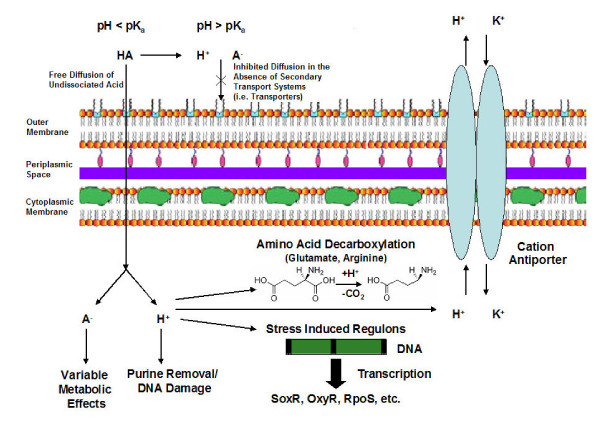
An overview of organic acid toxicity and tolerance mechanisms in *E. coli*. Diffusion of undissociated acid molecules can occur freely in acidic medium but is limited to transport systems at neutral or basic pH. The toxic effects associated with organic acids are the result of both anion specific affects on metabolism as well as increased internal proton concentrations. Affects on internal pH are mitigated by transport of protons out of the membrane, consumption of protons by decarboxylation reactions, and, more generally, induction of stress regulons. Anion specific tolerance mechanisms are not well characterized.

One of the primary factors contributing to the toxicity of organic acids is their ability to diffuse across *E. coli *cellular membranes when undissociated as opposed to the restricted passage of dissociated protons and anions (see Figure 2) [31,32]. Diffusion of dissociated acids is limited to secondary transport, which is known to involve H+/monocarboxylic acid symporters. However, the detailed mechanism and specificities of the transporters remain unknown [31]. *E. coli *maintain a cytoplasmic pH (pH_i _= 7.5) that is most often higher than that of the external media and typically well above the pKa of organic acids [44,45]. As a result, organic acids exist in the dissociated form within the cytoplasm. Thus, diffusing organic acids entering into the cytoplasm will dissociate and disrupt the pHi and anion pool of the cytoplasm. The resulting increase in internal acidity can affect the integrity of purine bases [46] and result in denaturing of essential enzymes inside the cell [35], both of which negatively affect cell viability.

Organic acid anions affect cell growth in a variety of manners. Increased anion concentration has been shown to lead to an increased transport of potassium ions into the cell, which increases turgor pressure [47,48]. To maintain a constant turgor pressure and cell volume, glutamate is transported out of the cell [48]. This transport activity concomitantly disrupts the osmolarity of the cytoplasm, which in turn lowers the cell's growth potential and viability. In addition to this general anion effect, there are also effects specific to each organic acid. It has been proposed that enzymes involved in protein synthesis are sensitive to a combination of two unrelated mechanisms, including the acidification of pHi and the formation of an anionic pool [35]. Although this finding implies that the organic inhibition due to the anion pool could be acid specific, the details describing this dual inhibition mechanism remain unclear. Kirkpatrick et al. reported proteins exhibiting increased expression in response to extracellular acetate [33]. Among these are the OppA transporter, RpoS regulon, several amino acid uptake proteins, DNA binding proteins, and extreme-acid preiplasmic chaperones. Interestingly, when formate was introduced in place of acetate the expression of the previously mentioned proteins was repressed, indicating that the response was anion specific. This finding introduces new challenges in addressing organic acid tolerance. Specifically, it highlights the need to engineer both pH and as well as specific anion tolerance into host organisms.

Finally, production of organic acids might include intermediates that are themselves toxic. For example, 3-hydroxypropionic acid (3HP) is closely related to the antimicrobial compound Reuterin. Reuterin describes the hydroxypropionaldehyde (HPA) system including HPA, HPA dimer, and HPA hydrate. Reuterin is inhibitory to several bacteria, including *E. coli*, at concentrations as low as 0.03–0.05 g/L [49-51]. It is thought that the toxicity could be the result of inhibition of DNA synthesis [52]. It has been postulated that the reactivity of the aldehyde group of HPA causes DNA damage similarly to formaldehyde, which is the aldehyde analog of formic acid [49]. Intermediate toxicity can be managed either by optimization of the production pathway in the host or by engineering tolerance to the intermediate itself.

### Organic Acid Tolerance in *E. coli*

*E. coli *has a remarkable ability to remain viable under a broad range of pH conditions. This ability is essential for its survival in the mammalian digestive system where pH can vary between pH = 2–8. Several different acid tolerance mechanisms have been identified in *E. coli*. While each mechanism is capable of providing some degree of tolerance, they are regulated differently and confer varying levels of tolerance.

Although most acid tolerance systems are activated in stationary phase, acid tolerance as low as pH = 3 has been observed in exponential phase *E. coli *grown under aerobic conditions, which is advantageous from a productivity standpoint [30]. Although the underlying tolerance mechanism is not known, such tolerance can be reliably activated by adapting cells at sublethal pH values between 4.3 and 5.8 [53]. *E. coli *that exhibit growth phase tolerance remain viable at pH values on the same order as stationary phase tolerance, however the percent survival is significantly lower. Lin et al. reported 1% survival of the original culture following acid adaption at pH 4.3 followed by acid challenge at pH 3.3 compared to 0.0001% survival for unadapted cultures. This is compared to stationary-phase cultures, which exhibited up to 50% survival.

Three stationary phase acid resistance systems have been studied in the most detail [29,30]. These systems confer the highest levels of tolerance and are believed to be responsible for stationary phase *E. coli *survival when passing through the mammalian stomach. Acid resistance system 1 (AR1) is activated in slightly acidic media (pH 5.5) in the absence of extracellular glucose or amino acids. *E. coli *grown aerobically under these conditions retain viability under acid challenges as low as pH = 2.5 [54]. This system is also referred to as the oxidative or glucose-repressed system, since the expression of this system is thought to be regulated either directly or indirectly by RpoS and cyclicAMP receptor protein (CRP) [55,56]. Acid resistance system 2 (AR2) is activated in *E. coli *grown in aerobic conditions in acidic complex media. This system requires the presence of extracellular glucose and glutamate and is dependent upon genes encoding glutamate decarboxylase (*gadAB*) and a glutamate:GABA antiporter (*gabC*) [30]. Under such conditions, *E. coli *have been demonstrated to exhibit acidic resistance up to a pH of 2. The mechanism involves the expenditure of excess cytoplasmic protons during amino acid decarboxylation reactions (see Figure 2), thus raising the internal pH [54,55]. Acid resistance system 3 (AR3) parallels the mechanisms of AR2 with several slight deviations [30,54,55]. AR3 is activated under anaerobic conditions, in complex media with added glucose. It also involves amino acid decarboxylation reactions to lower the internal pH, but requires extracellular arginine in place of glutamate. AR3 also requires increased expression of arginine decarboxylase and an arginine: agmatine antiporter for increased acid tolerance.

Finally, several general acid tolerance mechanisms that regulate the physical properties of the membrane or the effectiveness of ion transport have been identified. These active responses, or those that occur as a result of the cell's ability to sense pH changes, are independent of growth and are induced by pH shifts as small as 0.2 pH units [57]. The first response is the ability of the microorganism to adjust membrane properties, such as lipid content, thus effectively changing the proton permeability [57]. Another cellular response to acid shock is the induction of genes responsible for repairing and preventing lethal cellular damage. Specifically, increased expression of the *oxyR *and *soxR *regulatory genes has been observed by transcriptional profiling of acid tolerant phenotypes [45,58]. These systems regulate the removal of damaging oxidizing agents, thus preventing further DNA damage under acidic stress [46]. Finally, acid tolerance can be achieved by adjusting the ionic transporter efficiency, effectively regulating the anion and cation balance as a means of maintaining a constant internal pH [47].

### Organic Acid Production in *E. coli*

Metabolic and genetic engineering, directed evolution, and classic strain selection have all been employed in the development of *E. coli *strains that produce building block organic acids, including lactic-acid, succinic acid, and 3HP [17,25,59,60]. Improved titers have been achieved due to optimization of fermentation conditions and relavant pathways utilized. However, titer limitations exist when fermentation is carried out in unbuffered media, which allows the pH to acidify due to increased acid concentration. Alternatively large amounts of base titrant are required to raise the pH of the media during the organic acid production leaving the final acid molecule in the undissociated form. Following production under these conditions, large volumes of acid must be added to recover the acid in the protonated form. Metabolic and genetic engineering of acid tolerance into production strains, making fermentation at a pH less than the pKa of the acid produced possible, would circumvent the need for the additional consumption of acid and base titrants, and thus lower the overall production cost. Similarly, engineering strain fitness to increase productivity at a decreased pH would improve productivity and reduce base consumption.

Lactic acid production is one of the most successful examples to date of the engineering of large volume chemical production in *E. coli*. *E. coli *was selected as a favorable host strain due to its ability to consume both pentose and hexose sugars and to generate optically pure L-lactic acid, which is the desired product for downstream polylactic acid (PLA) production [61,62]. An effective lactic acid producing strain of *E. coli *was created by induced expression of the L-specific lactic acid dehydrogenase (LDH) gene from *Streptococcus bovis*. High titers (50–75 g/L) were observed under controlled pH (pH = 7) and anaerobic conditions. Titers were drastically decreased (10–20 g/L) as the pH was allowed to drop with increasing acid production [59]. However, allowing the pH to fall below the pKa of lactic acid also resulted in decreased concentration of the acid in the undissociated form, which facilitated the subsequent isolation of the protonated acid. Interestingly, the choice of host strain made a significant difference in lactic acid production [59]. Those constructed from an *E. coli *B strain showed a titer of almost twice that produced from K12 derivatives. The increased production was attributed primarily to differences in the native growth characteristics rather than increased acid tolerance.

Economically competitive titers of succinic acid have also been achieved in *E. coli*. Strains were engineered to limit flux to other anaerobic byproducts normally formed during fermentation [60]. Specifically, succinic acid production was optimized by redirecting the metabolic flux at the pyruvate node away from lactate and formate through inactivation of the pyruvate-formatelyase and lactate dehydrogenase [60,63]. The maximum yield in succinic acid production was approximately 50 g/L in pH controlled cultures. However, similar to lactic acid studies, succinic acid production was significantly repressed when pH was not kept at neutral levels.

A final example of metabolic engineering organic acid production in *E. coli *was reported by Cargill in 2001 [17]. Suthers and Cameron engineered a 2-step glycerol to 3HP pathway in *E. coli*. Glycerol was first converted to 3HPA via a glycerol dehydratase enzyme (*dhaB *– isolated from *Klebsiella pneumoniae*). 3HPA was then converted to 3HP via an aldehyde dehydrogenase (*ald*). This first pathway was not ideal for several reasons including a very low reported titer (0.2 g/L), the use of the more expensive glycerol as opposed to glucose, and the generation of the highly toxic 3-HPA (reuterin) compound. Selifinova et al. later proposed five additional pathways for the production of 3-HP directly from glucose in *E. coli *[36]. Results for each of such pathways have yet to be reported. One issue that has yet to be addressed is how to fulfill the desire to produce 3-HP at a pH below the pKa = 4.51 of 3-HP, which would lessen the dependency on large volumes of base titrant to retain neutral pH at high titers.

Metabolic engineering of *E. coli *organic acid tolerance represents an important future opportunity. As discussed above, *E. coli *possess several systems for surviving pH as low as 2.0, which is much lower than what is required for an economical biorefining process. Since induction of these systems is well characterized and the relevant genes are known in many cases, future efforts might be better focused on the development of multi-stage fermentations that allow for generation of biomass prior to induction of acid tolerance and, ultimately, acid production. Future genetic engineering efforts might focus on engineering tolerance against the less well characterized metabolic effects associated with increased organic acid anion concentrations. For example, the addition of acetate, benzoate, and propionate to culture media at a concentration of 8 mM has been observed to inhibit growth of *E. coli *up to 50% [35]. The acetate inhibition is thought to be caused by limited methionine pools combined with increasing concentrations of homocysteine, a toxic intermediate, due to inactivation of a key enzyme in the methionine synthesis pathway, which can be countered by the addition of methionine to the media. This finding established that growth inhibition is the result of both of lowered pH and specific anionic effects, which decreases the activity of key enzymes. Thus, engineering tolerance to specific organic acid anion effects by increased expression of inhibited enzymes could aid in increasing overall process productivity.

## Conclusion

Organic acids are a valuable sector of the industrial chemical market, which have already been successfully produced through microbial fermentation. However, product titers have been variable, ranging from less than 1 g/L to concentrations cost competitive with current petrochemical production processes. These fermentation processes have been limited in *E. coli *due to product and intermediate toxicity. Toxicity is directly measured by growth inhibition, which specifically decreases productivity. This review highlighted what is known about organic-acid toxicity and tolerance mechanisms in *E. coli*. Specifically, *E. coli *are growth inhibited by the increase in both proton and associated anion concentrations that are characteristic of organic-acid production processes. While several acid-tolerance mechanisms have been characterized in *E. coli*, anion specific mechanisms require additional study. Thus, future metabolic engineering efforts that seek to improve understanding of these issues within the context of organic-acid biorefining applications should prove useful.

## Supplementary Material

Additional File 1 **Table 1: **Organic acids for platform biorefining applications. (* see references [64,65]) Click here for file
