# Supplementary figures and images for: Organic acid toxicity, tolerance, and production in Escherichia coli biorefining applications
Source: Microb Cell Fact. 2005 Aug 25;4:25. doi: 10.1186/1475-2859-4-25 (PMC1208944; doi:10.1186/1475-2859-4-25)

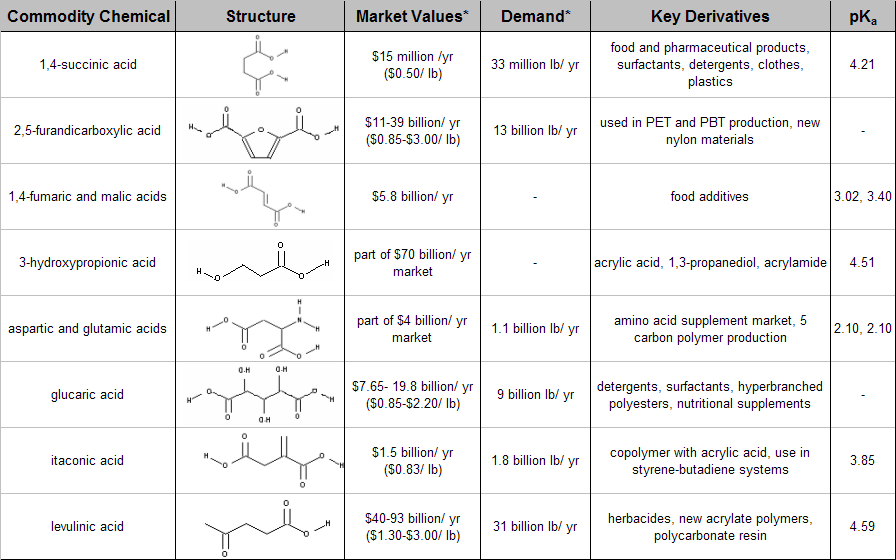

Supplement: Additional File 1 — Table 1: Organic acids for platform biorefining applications. (* see references [64,65]) [file 1475-2859-4-25-S1.doc]
